# Special Issue: Ribosome-Inactivating Proteins—Commemorative Issue in Honor of Professor Fiorenzo Stirpe

**DOI:** 10.3390/molecules22020316

**Published:** 2017-02-18

**Authors:** Els J.M. Van Damme

**Affiliations:** Department of Molecular Biotechnology, Faculty of Bioscience Engineering, Ghent University, 9000 Ghent, Belgium; ElsJM.VanDamme@UGent.be

The family of ribosome-inactivating proteins (RIPs) groups all enzymes (EC.3.2.2.22) with a so-called RIP domain which comprises *N*-glycosidase activity and enables these proteins to catalytically inactivate ribosomes. Ever since the discovery of the first ribosome-inactivating protein, this group of plant toxins has received a lot of attention from researchers in various fields. Ribosome-inactivating proteins have been studied in detail for their distribution in nature, their molecular structure and toxicity, and have proven valuable tools for agricultural and biomedical research. Despite their long history ribosome-inactivating proteins are still at the forefront of research on applicability of plant toxins.

This Special Issue of *Molecules* intended to collect state-of-the-art original research papers and review articles that cover the physiological importance of ribosome-inactivating proteins as well as their applications. Furthermore this Special Issue was to honour Professor Emeritus Fiorenzo Stirpe for pioneering work related to the study of ribosome-inactivating proteins from plants and the preparation of immunotoxins. The papers published in this Special Issue include four review papers and four original research articles.

The first review by Bolognesi et al. [1] summarizes the most important steps in the academic career of Prof. Stirpe and focuses on his contributions to the frontline research on ribosome-inactivating proteins over a period of 55 years. Together with his co-workers, Prof. Stirpe identified, purified and characterized a large number of new plant toxins. In addition, they studied in detail the biological properties and mode of action of several proteins, which ultimately gave rise to the name “ribosome-inactivating proteins” to designate these toxic plant proteins. The ultimate aim of the research was the therapeutic application of ribosome-inactivating proteins as valuable tools for medical research, in particular the preparation of immunotoxins for cancer research. Because of his outstanding contribution to the field of immunotoxins and their applications in experimental therapy, Prof. Stirpe received several prestigious prizes. In a second review, Bolognesi et al. [2] provide an historical overview of ribosome-inactivating proteins from plants. The history of these toxins started with the early discovery of ricin and abrin at the end of the eighteenth century, their purification and characterization of the biological activities at biochemical level, but has since evolved towards a multidisciplinary research, leading to applications in agriculture and biomedicine.

Knowledge about the mode of action of ribosome-inactivating proteins is crucial to investigate possible applications. In their review, Shi et al. [3] studied the structural basis for the interaction of ribosome-inactivating proteins with ribosomes. Although the target site of ribosome-inactivating proteins is a specific adenine residue in the RNA, ribosomal proteins also contribute to the catalytic efficiency of the enzymes. The authors suggest that the binding to these ribosomal proteins is crucial for the specificity of ribosome-inactivating proteins towards eukaryotic and prokaryotic ribosomes. 

The review written by Polito et al. [4], focuses on ribosome-inactivating proteins from plants and their use in traditional medicine. Because of their toxic properties many RIP containing plants are used in folk medicine worldwide, unfortunately often without scientific validation. One of the most promising prospects for ribosome-inactivating proteins is their use as part of immunotoxins that recognize tumour cells. In view of applications as anti-cancer agents, it is important that the selectivity, immunogenicity, half-life and toxicity of RIP-based conjugates are investigated. Therefore Chooniedass et al. [5] studied the engineering and biological activity of different conjugates containing the plant toxin deBouganin for their interaction with a panel of breast cancer cell lines. In an attempt to adapt ribosome-inactivating proteins for clinical use, Sun et al. [6] investigated the production, identification and anti-tumour activity of mono-pegylated ribosome-inactivating proteins from *Momordica charantia*.

Ribosome-inactivating proteins are widely distributed in flowering plants. Shang et al. [7] report on the occurrence of ribosome-inactivating proteins in the family Rosaceae. They retrieved multiple RIP sequences from the genomes of *Malus domestica*, *Prunus persica*, *Prunus mume*, *Pyrus communis* and *Pyrus brestschneideri* but not from the genomes of *Fragaria vesca* and *Fragaria ananassa*. The results of in silico analysis and molecular modelling revealed that the amino acids known to be important for the activity of ricin are highly conserved in the active site of the RIP sequences from *Malus domestica*, suggesting that these ribosome-inactivating proteins are functional proteins, as also inferred from data of translation inhibition experiments with recombinant ribosome-inactivating proteins from apple.

Extracts from different elderberry (*Sambucus nigra*) tissues as well as elderberry juices are a rich source of lectins, ribosome-inactivating proteins and low molecular weight nutraceuticals. Jiminez et al. [8] report that short-time heat treatment of extracts reduced potential allergy-related risks derived from elderberry consumption, but did not compromise the anti-oxidant properties and free-radical scavenging activities. Future in vivo studies will have to address questions related to lectin digestibility in animals and in food matrices.

This Special Issue provides the readers with several up-to-date overview papers and original research articles that highlight the state-of-the-art of research on ribosome-inactivating proteins and their prospects for applications.
